# The Quanity of Drugs Used

**Published:** 1877-07

**Authors:** 


### THE QUANTITY OF DRUGS USED.

At the meeting of the American Pharmaceutical Asssociation this fall, it was stated that the total imports for the fiscal year were $476,000,000, while the total exports were $596,000,000, leaving a balance in our favor of $120,000,000.

The character of many of the powdered drugs of the market was alluded to, and their quality hinted at by the comparative prices at which they were sold and the value of good crude drugs. Borax was exported to the extent of 3,000,000 pounds, while California and Nevada have produced in all during the year 6,000,000.

The Pacific coast has furnished during the year 54,000 flasks of mercury, while less than that has been produced by all the other nations of the world combined. The product of opium was stated to be smaller in amount for the year than was anticipated and it would probably advance in price still further.—*Medical and Surgical Reporter.*

